# The Origin of Efficiency in III‐Nitride Micro‐Light‐Emitting Diodes

**DOI:** 10.1002/advs.202520738

**Published:** 2026-04-02

**Authors:** Jeong‐Hwan Park, Markus Pristovsek, Dong‐Pyo Han, Jiwon Kim, Jong‐In Shim, Bumjoon Kim, Soo Min Lee, Drew Hanser, Dong‐Seon Lee, Yoshio Honda, Tae‐Yeon Seong, Hiroshi Amano

**Affiliations:** ^1^ Institute of Materials and Systems for Sustainability Nagoya University Nagoya Japan; ^2^ MicroLED Development Team Samsung Electronics Giheung‐gu Gyeonggi‐do South Korea; ^3^ Display & Semiconductor Engineering School of Electrical Engineering Pukyong National University Busan South Korea; ^4^ Department of Photonics and Nanoelectornics Hanyang University Ansan Gyeonggi‐do South Korea; ^5^ Veeco Instruments Inc. Somerset New Jersey USA; ^6^ Department of Semiconductor Engineering Gwangju Institute of Science and Technology Gwangju South Korea; ^7^ Department of Materials Science and Engineering Korea University Seoul South Korea

**Keywords:** bulk material, diffusion length, external quantum efficiency, microLED, wavelength

## Abstract

We demonstrate that the primary factor determining the external quantum efficiency (EQE) of InGaN‐based micro‐scale light‐emitting diodes (µLEDs) depends on their internal state. A comparative photoluminescence (PL) study shows that the lateral diffusion length of carriers in InGaN red µLEDs is significantly shorter than in InGaN blue µLEDs, primarily due to inhomogeneity in the bulk material. This results in an insignificant change in PL intensity regardless of sidewall conditions. Additionally, examinations of EQE and peak wavelength across various epitaxial designs and sidewall conditions reveal that sidewall‐surface recombination does not significantly impact EQE in InGaN red µLEDs. Meanwhile, the peak wavelength, which represents the radiative recombination rate given by the quantum well design of InGaN red µLEDs, is found to dominantly influence the EQE of InGaN red µLEDs. Furthermore, statistical analysis based on the relative standard deviation indicates that the peak wavelength is one of the primary determinants of EQE in InGaN red µLEDs. These findings suggest that addressing internal state is crucial for optimizing EQE of µLEDs.

## Graphical abstract

The images show panchromatic cathodoluminescence of III‐V materials, which reveal that AlGaInP red wafers, having fewer defects, support longer carrier diffusion lengths. Meanwhile, the density of defects increases with longer emission wavelengths in the InGaN system, illustrating how generated carriers pass through the material and how defects limit their effective movement.

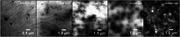

## 1 Introduction

**FIGURE 1:**
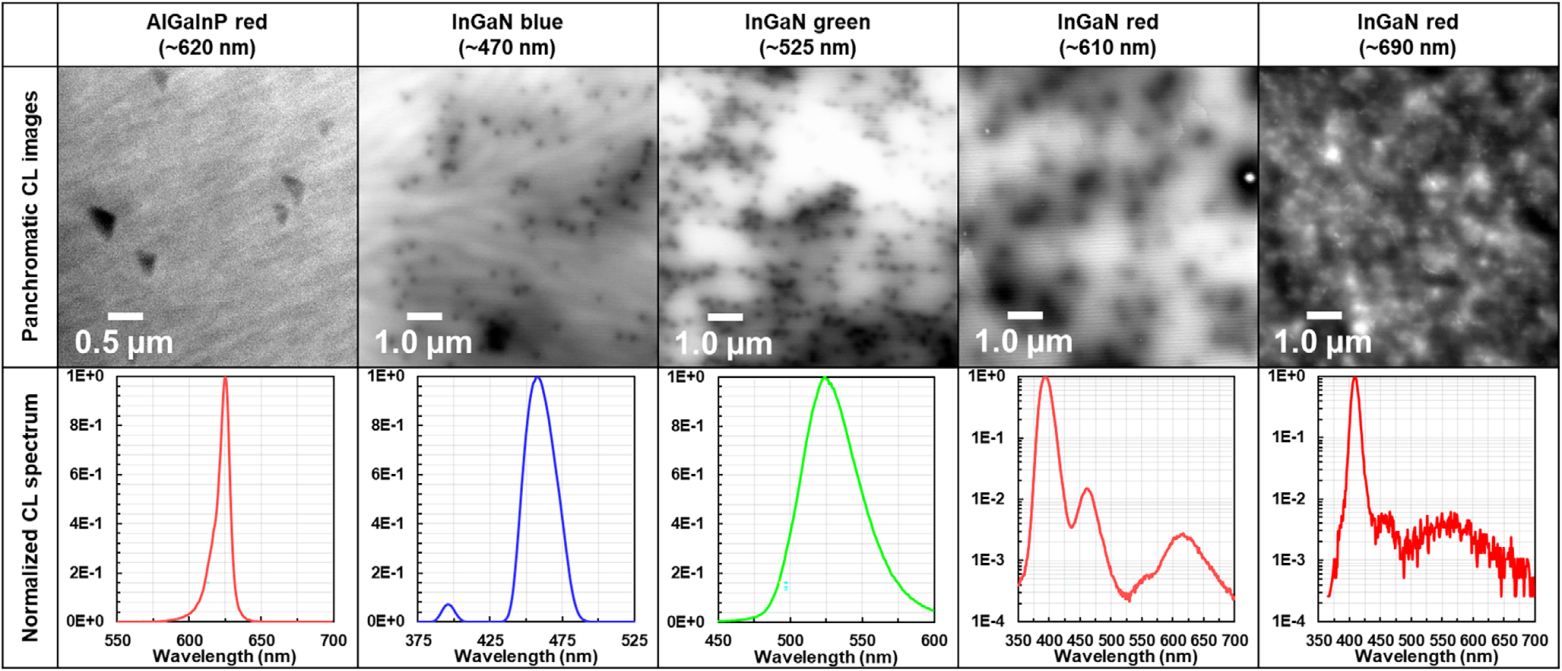
Example of panchromatic CL images and spectrum for AlGaInP red, InGaN blue, green, ∼610, and ∼690 nm red, showing their difference in defect levels and inhomogeneity. All images were obtained at Nagoya University, Amano laboratory.

Understanding the degradation mechanisms in InGaN‐based micro‐light‐emitting diodes (µLEDs) is crucial for applications in next‐generation displays, such as augmented reality (AR), which require ultra‐small chips with high efficiency (e.g., less than 2.0 µm chips for over 10,000 PPI) [1]. However, reducing chip size often leads to efficiency loss, posing a significant obstacle to the commercialization of µLEDs for AR due to insufficient device performance. Over the past decade, much research has focused on this issue, primarily attributing efficiency degradation to sidewall‐surface states induced by plasma‐based dry etching and the intrinsic surface states [2]. In addition, solutions overcoming the problem have also been developed, focusing on the sidewall‐surface state [3]. Notably, the EQE of AlGaInP red µLEDs shows dramatic size‐dependent degradation, over 75% reduction when scaled from 160 to 10 µm, while InGaN red µLEDs exhibit relatively independent EQE with respect to size and sidewall surface states [4, 5, 6]. This contrast fundamentally stems from differences in their internal material states, specifically the inhomogeneity within the active region. We present a series of panchromatic cathodoluminescence (CL) images of III–V materials emitting visible colors (Figure 1), which reveal that AlGaInP red wafers, having fewer defects, support longer carrier diffusion lengths [7]. Conversely, the density of defects (and dark areas indicating inhomogeneity) increases with longer emission wavelengths in the InGaN system. These CL images illustrate how generated carriers pass through the material and how defects limit their effective movement. Specifically, defect‐induced inhomogeneity predominantly causes carriers to fall into defect sites, reducing the number of carriers reaching the sidewall surface [8, 9]. This raises the question of which defect type, bulk defects or sidewall surface defects, is more detrimental to ultra‐small red µLEDs operating under real‐world conditions, particularly considering the longer carrier diffusion length in AlGaInP compared to InGaN [10, 11, 12]. While the primary mechanisms behind the efficiency degradation in InGaN blue and AlGaInP red µLEDs are well‐studied, comprehensive investigations into which parameters primarily determine the EQE of InGaN red µLEDs remain limited. Furthermore, the inhomogeneity issue in InGaN red µLEDs, which impairs device uniformity, continues to hinder large‐scale, high‐performance applications. Although the main mechanisms determining the EQE degradation of InGaN blue and AlGaInP red µLEDs have been investigated, studies on the parameters that primarily determine the EQE of InGaN red µLEDs have not been investigated in detail. Furthermore, the inhomogeneity issue of InGaN red µLEDs, which impedes the high uniformity among operating devices, remains. Thus, this study aims to clarify the origin determining the efficiency of InGaN‐based µLEDs by investigating their internal state within the framework of carrier dynamics, specifically focusing on the lateral carrier diffusion length and the radiative recombination rate.

First, we investigate the possibility that sidewall‐surface recombination may be negligible in devices with short lateral carrier diffusion length (λ). This is examined by comparing InGaN blue and red µLEDs under various sidewall conditions using simple photoluminescence (PL) experiments. Second, we demonstrate that the primary factor influencing the EQE of InGaN red µLEDs is closely related to their peak wavelength, which reflects the radiative recombination rate (*B*), as investigated through electroluminescence (EL). Additionally, we perform statistical analyses to understand the relationship between EQE and peak wavelength, revealing that inhomogeneity effects diminish with increasing current density. To clearly distinguish between the two experiments, the details are summarized in Tables 1 and 2. Since the fabrication processes for each experiment were identical, we assume that light extraction efficiency plays an insignificant role in the present investigation. The same epitaxial wafer was used in both groups #2 and #3.

**TABLE 1:** A summary of PL characteristics of 25 µm diameter blue and red µLEDs.

Group	TMAH treatment time (second)	Intensity (median)	Wavelength (median)	Estimated mapping count
#1	0	0.39	459.7	417
	30	0.90	460.0	468
	60	1.00	460.0	462
#2	0	0.97	594.5	477
	30	1.00	594.3	468
	60	0.95	592.0	435

**TABLE 2:** A summary of the device structure and characteristics for EL experiments.

Group	QW thickness (nm)	QW indium content (%)	Mesa structure (µm^2^)	TMAH treatment time (min)	ALD‐Al_2_O_3_ thickness (nm)	PECVD‐SiO_2_ thickness (nm)	Estimated pixel number	Peak wavelength at 1 A/cm^2^ (median)	J_peak_ (A/cm^2^) (median)	EQE_peak_ (%) (median)
#3	2.7	30	80 × 5	0	10	400	17	608 nm	16.6	0.685
				10			15	612 nm	16.6	0.657
#4	8.3	13	40 × 40	0			18	696 nm	5.0	0.241
				10			19	686 nm	5.0	0.260

## 2 Results and Discussion

### 2.1 Impact of Diffusion Length on InGaN µLEDs

**FIGURE 2:**
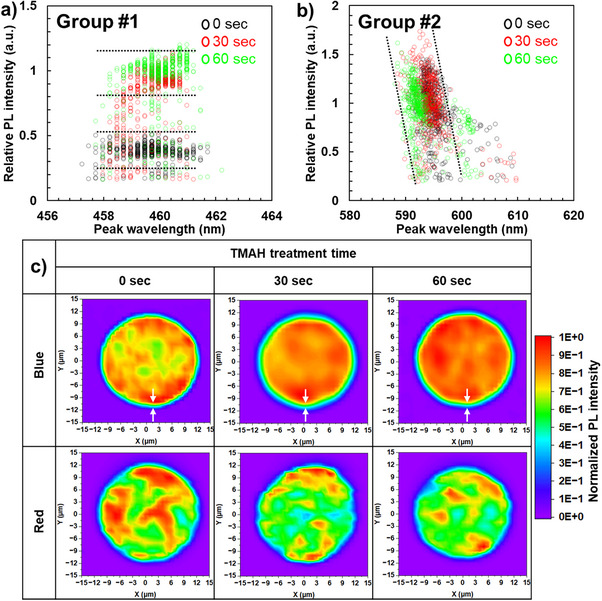
Peak wavelength‐dependent PL intensities obtained from 25 µm diameter µLEDs are mapped to data for (a) blue (group #1) and (b) red µLEDs (group #2) following various TMAH treatment times. (c) Normalized PL intensity mapping images for blue and red µLEDs following TMAH for 0, 30, and 60 s.

It is important to recognize that sidewall damage does not affect blue and red µLEDs equally. To investigate this, PL intensity was plotted as a function of the peak wavelength, enabling us to quantitatively assess the effects of TMAH treatment by the wavelength shifts. The experimental results and detailed measurement conditions are summarized in Table 1. Figure 2a,b shows PL intensity vs. peak wavelength for 25 µm diameter blue and red µLEDs. Quantitative mapping images of both peak wavelength and intensity are available in Figure S1. In blue µLEDs, the PL intensity remains relatively independent of the peak wavelength, a trend consistent regardless of TMAH treatment. Notably, PL intensity increases approximately 2.0–2.5 times after TMAH treatment. The data for treatment times of 30 and 60 s overlap significantly, indicating that TMAH's effect saturates after a certain duration. This suggests that a short TMAH treatment is sufficient for removing sidewall defects, which are typically only a few nanometers deep due to ICP‐RIE‐induced damage [13, 14, 15, 16, 17]. Additionally, recent studies showed that a TMAH treatment of 60 and 600 s results in similar EQE curves at low current densities [18]. This aligns with the observed increase in PL intensity after both 30 and 60 s of TMAH treatment. Therefore, one of the primary factors influencing sidewall‐surface recombination is the sidewall surface states. This highlights the importance of removing sidewall defects and supports the effectiveness of a short chemical treatment. If sidewall defects solely contribute to recombination, similar changes should be observed in red µLEDs. However, as shown in Figure 2b, the PL intensity of red µLEDs remains unchanged after TMAH treatment. This suggests that sidewall defects are not the primary factor affecting red µLED performance. Supporting this, the EQE of InGaN red µLEDs shows minimal dependence on chip size down to several micrometers, unlike blue and green µLEDs, indicating a reduced influence of sidewall recombination [19]. Based on this scenario, we define the total non‐radiative recombination rate, which predominantly determines EQE at low current densities, by incorporating the lateral diffusion length (λ) as an additional parameter:

(1) $$A=A_{0}+A_{s}\lambda \frac{l}{S}\;\left[s^{-1}\right]$$

where *A*_0_ [s^−1^] is the non‐radiative recombination rate in the bulk caused by dislocations, vacancies, etc.; *A_s_* [s^−1^] is the sidewall‐surface recombination rate associated with the surface states; λ [*m*] is the lateral diffusion length of carriers, enabling them to reach the sidewall; *l* [m] is the peripheral length of the device, and *S* [*m* ^2^] is the device area. The inclusion of λ and *A_s_*, representing bulk and surface effects, is based on our experimental findings, which compare InGaN blue and red µLEDs. First, experiments demonstrate that InGaN red µLEDs with high defect densities (∼10^9 ^cm^−2^) experience minimal size‐dependent degradation, indicating reduced sidewall‐surface recombination [20]. This occurs because the high defect density in the bulk limits carrier diffusion, resulting in a short λ [21]. Second, atom probe tomography (APT) measurements of InGaN red µLEDs with 30% indium content confirm that the absence of size‐dependent degradation stems from the short λ, which minimizes sidewall effects and inner‐state related issues [22, 23]. Overall, the negligible sidewall‐surface recombination in InGaN red µLEDs agrees with the observations in Figure 2b. Third, recent our observations showed that inserting a SiN layer between the first QW and n‐GaN, which shortens λ, reduces sidewall‐surface recombination [24]. Thus, λ is a critical parameter influencing the total non‐radiative recombination rate in µLEDs. Our studies and others in literature indicate that λ in blue µLEDs is on the order of micrometers [25, 26], whereas in red µLEDs it is significantly shorter, as evidenced by normalized PL mapping data. To determine the difference in λ between blue and red µLEDs, we analyzed the intensity distribution depending on TMAH treatment time (Figure 2c). The distribution in blue µLEDs is radially uniform, while the region with increased PL intensity for the red µLED are patchy, and some even close to the border, again confirming a shorter lateral diffusion carrier length in the red QWs. This appears to be affected by the peak wavelength. Specifically, the PL intensity of red µLEDs decreases as the peak wavelength increases (Figure 2b) which is very much consistent with localization at small regions at lower power which mostly get delocalized at the higher power of the PL mapping. In contrast, the blue µLEDs show relatively consistent intensity across wavelengths, because the carriers are not localized and can find favorable recombination spots no matter where excited (see dotted lines). This variability further underscores the different carrier diffusion behaviors between the two types of µLEDs. Next, we observed that the intensity distribution near the sidewall differs between blue and red µLEDs. As marked by white arrows (Figure 2c), blue µLEDs consistently display a circular band with a width of approximately 1 µm at half of the maximum intensity, aligning with previous findings and confirming the λ of blue µLEDs. In contrast, this behavior is not observed in red µLEDs, where the half‐maximum intensity is randomly distributed across the entire area. Additionally, the intensity distribution blue and red stays the same with and without TMAH treatment, suggesting that λ is not influenced by sidewall condition but is instead an inherent property of the QW, such as indium content or growth conditions (Figure 1) [25, 26]. Therefore, the disparity in λ between blue and red µLEDs supports the validity of Equation (1) in describing the main mechanism of SRH non‐radiative recombination affecting the EQE in µLEDs.

### 2.2 Impact of Peak Wavelength on InGaN Red µLEDs

**FIGURE 3:**
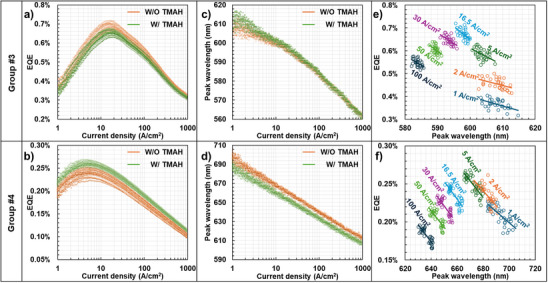
Demonstration that the EQE of InGaN red µLEDs depends primarily on the peak wavelength rather than the sidewall conditions, which correlates with the radiative recombination rate. EQE curves of (a) Group #3 and (b) Group #4, and peak wavelength curves of (c) Group #3 and (d) Group #4. Revealing a linearity between EQE and the peak wavelength observed at each current density (e) Group #3 and (f) Group #4.

PL studies indicate that InGaN red µLEDs are less affected by sidewall‐surface recombination, though their emission wavelength varies (Figure 2). To investigate this further, we compared InGaN red µLEDs emitting at relatively short (∼610 nm) and long (∼690 nm) wavelengths using EL measurements. These µLEDs had a very different inner structure, the group #3 µLEDs had continues QWs [23] while the group #4 µLEDs emission was mostly from isolated regions of less than 200 nm diameter [20]. The processing steps, including ICP‐RIE, TMAH treatment, passivation, and contact metallization, were consistent with our previous study [23]. Table 2 summarizes the sample characteristics, confirming their device structures, performance, and other relevant details. Figure 3a,b display EQE curves for each group. Notably, the effect of TMAH treatment varies between groups; for example, EQE improves in group #4 after TMAH, while it does not in group #3. This contrasts with the behavior observed in blue µLEDs (Figure 1), suggesting that reducing *A_s_* is ineffective for InGaN red µLEDs. Instead, another factor, such as a short λ caused by material inhomogeneity, could be responsible. The EQE can be modeled using the classical ABC model, as described below:

(2) $$EQE=IQE\times \;\eta_{e}=\frac{Bn^{2}}{An+Bn^{2}+Cn^{3}}\;\times \eta_{e}$$

*B* represents the radiative recombination rate [cm^3^/s], *C* is the Auger recombination rate [cm^6^/s], *n* is the carrier density [cm^−3^], and *η_e_* is the light extraction efficiency. If λ is too small to have a significant effect, the second term in Equation (1), which accounts for sidewall‐surface recombination, is negligible in the denominator of Equation (2). To understand this mathematically, we analyze *J_peak_* at the peak EQE by differentiating Equation (2).

(3) $$J_{peak}=e_{0}\;wA\left(\frac{B}{C}+2\sqrt{\frac{A}{C}}\right)\propto A_{0}+A_{s}\lambda \frac{l}{S}\;\;\left[A/cm^{2}\right]$$

Equation (3) confirms that *J_peak_* is proportional to *A_s_* and λ. However, as shown in Figure 3a,b, neither *J_peak_* nor the EQE curve changes significantly with TMAH treatment. These results suggest that λ in InGaN red µLEDs is negligibly small, rendering *A_s_* ineffective. Despite understanding λ’s minimal influence on EQE, the slight variations in EQEs among different groups remain unexplained. We hypothesize that this variability stems from inhomogeneities in the emission wavelength across each evaluatedy pixel, likely due to uniformity challenges in InGaN red epitaxy [27, 28, 29]. Figure 3c,d shows that all estimated pixels exhibit different peak wavelengths, confirming the inhomogeneity when using 4‐inch epitaxial wafers. Notably, the peak wavelength in Group #3, characterized by higher indium content (∼30%), becomes more inhomogeneous at lower current densities. Interestingly, within each group, samples with shorter peak wavelengths tend to have higher EQEs, indicating a correlation between wavelength uniformity and device performance. In other words, the dominant factor influencing the EQE of InGaN red µLEDs is not the sidewall condition but the peak wavelength. To illustrate this, we propose an example analyzing the relationship between EQE and peak wavelength at (Figure 3e,f). Data points, without distinguishing TMAH treatment, are collected at each current density. Overall, the EQE decreases gradually with increasing peak wavelength in both groups, showing a linearity, which is consistent with the PL results for red µLEDs shown in Figure 2b. This trend suggests that EQE variations are linked to the radiative recombination rate (*B*), which is governed by electron‐hole wavefunction overlap (Г_e‐hh_). Theoretical studies confirm that Г_e‐hh_ decreases as the peak wavelength increases, primarily due to stronger piezoelectric fields, resulting in smaller *B* within QW [30, 31, 32]. Specifically, the piezoelectric polarization in Group #4 is expected to be approximately 1.38 times larger than in Group #3, which could account for differences in EQE [33, 34]. Therefore, these results indicate that *B*, and thus the wavefunction overlap, plays a key role in determining the EQE of InGaN red µLEDs, while the inhomogeneity in peak wavelength reflects the uniformity or variability of the epitaxial wafer.

### 2.3 Revealing the Correlation Between EQE and Peak Wavelength via Statistics

Examining the data for EQE and peak wavelength reveals considerable dispersion within each group, likely due to factors such as non‐uniformities in QW thickness and indium content, affecting *B*. This variability allows for a statistical analysis to better understand the relationship between EQE and peak wavelength in InGaN red µLEDs. To this end, we employ a statistical method called the relative standard deviation (RSD) that characterizes data distribution with normalization, providing additional evidence that the EQE of InGaN red µLEDs primarily depends on the peak wavelength.

(4) $$\mathrm{RSD}\;=\frac{\mathrm{standard}\;\mathrm{deviation}}{\mathrm{median}}\;$$

**FIGURE 4:**
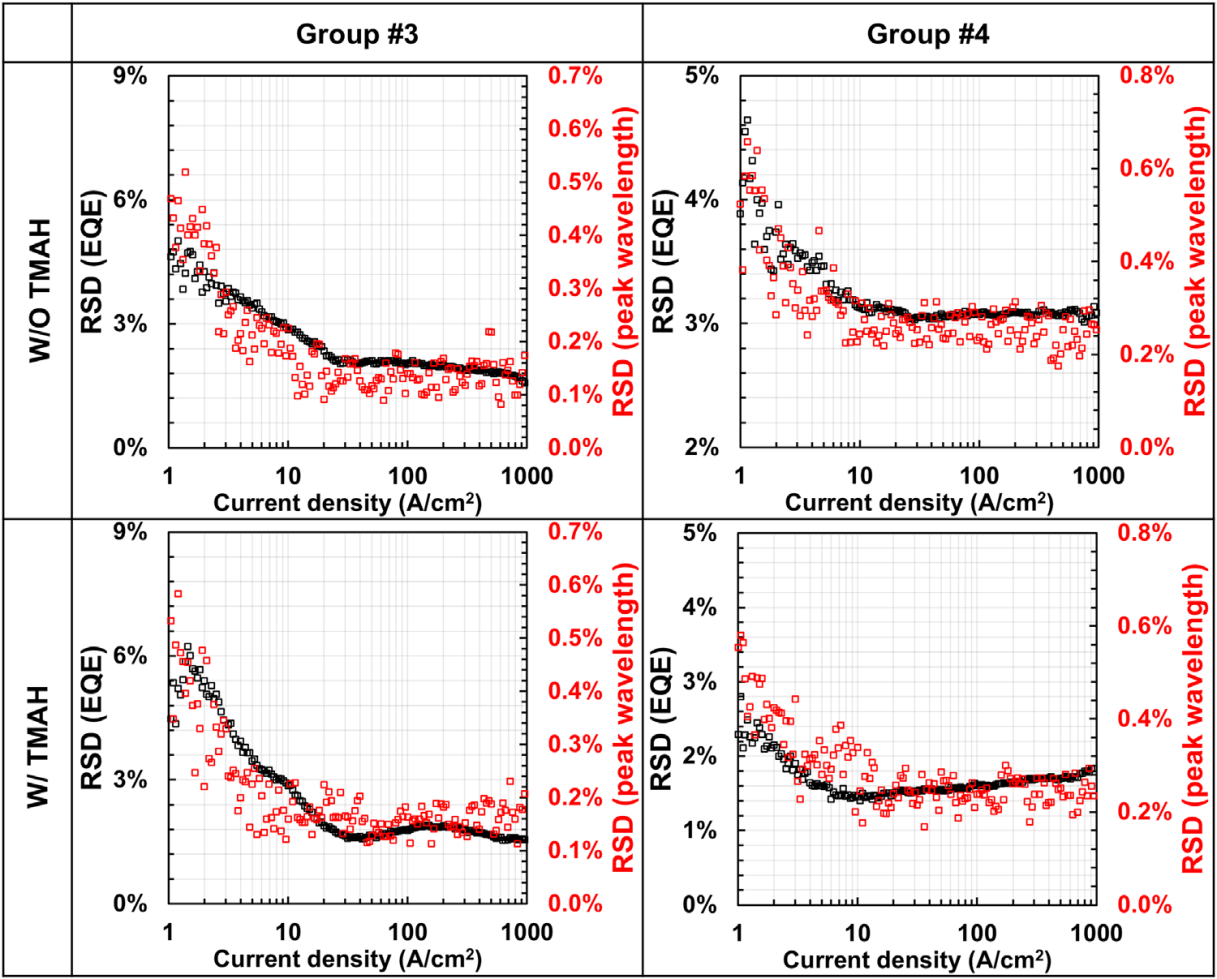
RSD of EQE and peak wavelength as a function of current density for each group. This statistically reveals that EQE of InGaN red µLEDs is a function of the peak wavelength.

Equation (4), a statistical model, elucidates the data distribution for each sample. By applying this equation, we confirmed the distribution patterns of EQE and peak wavelength across all samples. As shown in Figure 4, notably, RSDs of EQE and peak wavelength overlap significantly across the entire current density range and are saturated starting from ∼J_peak_ in each group. This strong similarity supports the hypothesis that the EQE of InGaN red µLEDs is primarily relevant to their peak wavelengths, as expected from the correctlation observed around the peak EQE from Figure 3e,f. This phenomenon is naturally understood by a conventional ABC model of Equation (2). 1) at low current density equaled to low carrier density, IQE is simply expressed to be $\frac{Bn}{A}$, which confirms the entire shape of RSD of EQE is primarily attributed to *B* where the denominator is assumed constant. However, with increasing current density, the denominator of IQE becomes large (e.g., *An* + *Bn* ^2^ + *Cn* ^3^), which results in a relatively weak in the numerator of *Bn* ^2^. Thus, RSD is exponentially decreasing by current density. 2) All graphs are saturated starting from near J_peak_. From this stage, *C* could start to be a dominant factor. 3) at high current density, IQE can be expressed to $\frac{B}{B+Cn}$, which implies that overall dispersion by current density could be relevant to *C*. If the dispersion of *C* is significant, RSD would change as the current density increases. 4) as such, RSD at high current density is interpreted as the dispersion of *C* is unexpectivly small, rendering saturated RSD by *B*. Based on interpreation, it suggests that EQE of InGaN red µLEDs is a function of the peak wavelength, reflecting *B*. As a result, the efficiency of InGaN red µLEDs is less influenced by sidewall effects but governed by *B* overall primarily at a certain current density. Therefore, a viable route to high‐efficiency InGaN red µLEDs involves developing epitaxial designs that enhance *B*, without significantly altering the peak wavelength [35, 36, 37, 38]. Furthermore, the saturation of RSD at higher current densities provides insights into epitaxial uniformity challenges. Addressing this issue by optimizing device operation at higher current densities could improve uniformity and enable better performance for AR display applications.

## 3 Conclusion

In conclusion, we systematically elucidated that the dominant factor governing the efficiency of InGaN µLEDs is their internal material state. PL intensity in blue µLEDs varies significantly even with a short TMAH treatment of 30 s, while red µLEDs show minimal changes. The normalized PL intensity at the edge of blue µLEDs is less than half of that in a 1 µm round band, whereas the intensity distribution in red µLEDs appears random regardless of TMAH treatment, indicating differences in carrier diffusion lengths. Comparative studies with different epitaxial designs revealed that in red µLEDs, sidewall conditions have a negligible impact on EQE due to their short diffusion lengths, whereas the peak wavelength, reflecting the radiative recombination rate, plays a critical role. Furthermore, statistical analysis via RSD that the relationship between EQE and peak wavelength remains consistent across the entire current density range, demonstrating that the efficiency of InGaN red µLEDs predominantly depends on their peak wavelength, reflecting the radiative recombination rate.

## 4 Experimental Section

### 4.1 CL Experiment

To illustrate the status of defect and inhomogeneity for AlGaInP red (∼630 nm), InGaN blue (∼470 nm), InGaN green (∼525 nm), and InGaN red (∼610 nm and ∼690 nm), panchromatic CL was conducted using Gatan Mono CL with Hitachi SEM S4300 over 10keV accelating voltage. Bracket indicates a peak wavelength using the electrical injection (e.g., EL).

### 4.2 PL Experiment

InGaN based blue and red epitaxial LED wafers grown on sapphire substrate, which have 2.5 nm thick In_0.155_Ga_0.845_N and 2.7 nm thick In_0.3_Ga_0.7_N quantum well layers, respectively, were dry etched by a typical ICP‐RIE (RIE‐200iPN‐2, SAMCO) to open up n‐GaN layer. Where an ICP power and bias power were 150 and 15 W, respectively, with Cl_2_ of 300 sccm. Subsequently, 25wt. % tetramethylammonium hydroxide (TMAH) treatment was conducted at 80°C for 0, 30, and 60 s. To proceed with the PL experiment, 355 and 405 nm laser diodes were used for blue and red LEDs, respectively, where the measurement interval was 1 µm.

### 4.3 EL Experiment

Two InGaN red epitaxial LED wafers grown on sapphire substrate were chosen to confirm the negligible sidewall‐surface recombination on InGaN red µLEDs. Each wafer has a 2.7 nm thick In_0.3_Ga_0.7_N and an 8.3 nm thick In_0.13_Ga_0.87_N quantum well layer [19, 22]. To fabricate devices, a 100 nm indium tin oxide (ITO) layer was deposited as a p‐contact layer. Next, the dry etching process using ICP‐RIE was conducted to reveal n‐GaN layer (same as the above PL experiment). N‐pad layer of Cr/Au (30/200 nm) was deposited. After, 25wt. % tetramethylammonium hydroxide (TMAH) treatment was conducted at 80°C for 0 and 10 min to distinguish the sidewall‐surface state. Subsequently, a 10 nm thick Al_2_O_3_ layer and a 400 nm SiO_2_ layer were deposited using ALD (AD‐100LE, SAMCO) and PECVD (PD‐220NL‐2, SAMCO), respectively. To create a P‐pad layer, CF_4_ based RIE (RIE‐10NR, SAMCO) etched Al_2_O_3_ and SiO_2_ and Al/Ni/Au (500/10/200 nm) was deposited. The entire device fabrication process was the same as reference 22. The light output for obtaining peak wavelength and EQE was measured using an integrating sphere (FOIS‐1, Ocean Insight) connected to a spectrometer (FLAME‐S, Ocean Insight).

### 4.4 Statistical Analysis

To plot the RSD of EQE and peak wavelength for each group, we first adopted the sample size (n) from the estimated pixel number described in Table 2. After, the standard deviation and median for EQE and peak wavelength were evaluated in each group. The calculated standard deviation, median, and p‐value as a function of current density is available in Figure S2.

## Conflicts of Interest

The authors declare no conflict of interest.

## Supporting information




**Supporting File**: advs74282‐sup‐0001‐SuppMat.docx.

## Data Availability

The data that support the findings of this study are available from the corresponding author upon reasonable request.
