# Supplementary material for: The Origin of Efficiency in III‐Nitride Micro‐Light‐Emitting Diodes
Source: Adv Sci (Weinh). 2026 Apr 2;13(25):e20738. doi: 10.1002/advs.202520738 (PMC13137796; doi:10.1002/advs.202520738)
Supplement: Supplementary file 1 — Supporting File: advs74282‐sup‐0001‐SuppMat.docx. [file ADVS-13-e20738-s001.docx]

**Supplementary information**

**
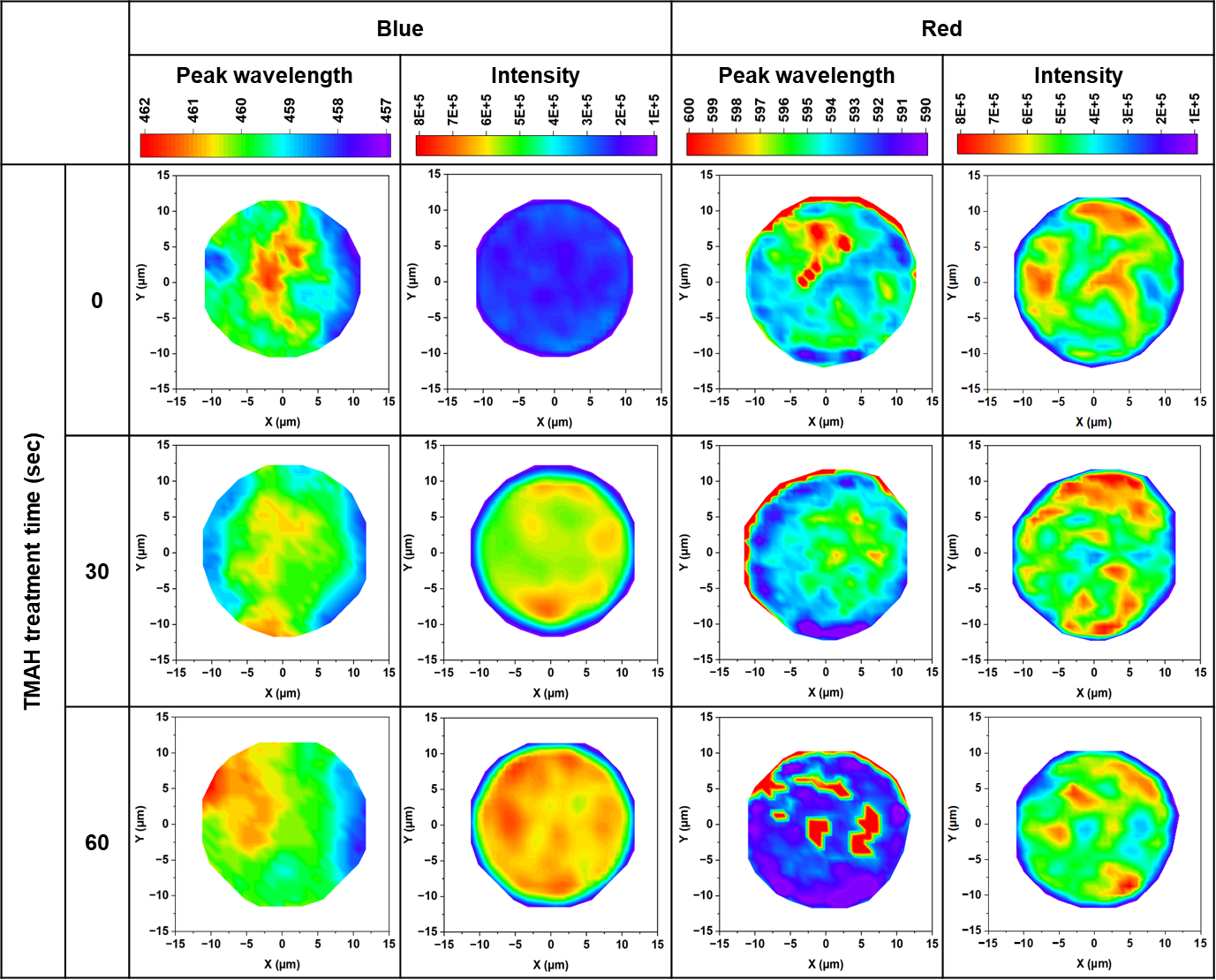
**

Figure S1. PL mapping images of peak wavelength and intensity for InGaN blue and red LEDs with varying TMAH treatment time. This result implies that the intensity of blue LED is dominantly influenced by TMAH treatment time, whereas that of red LED depends on the peak wavelength, regardless of TMAH treatment time.


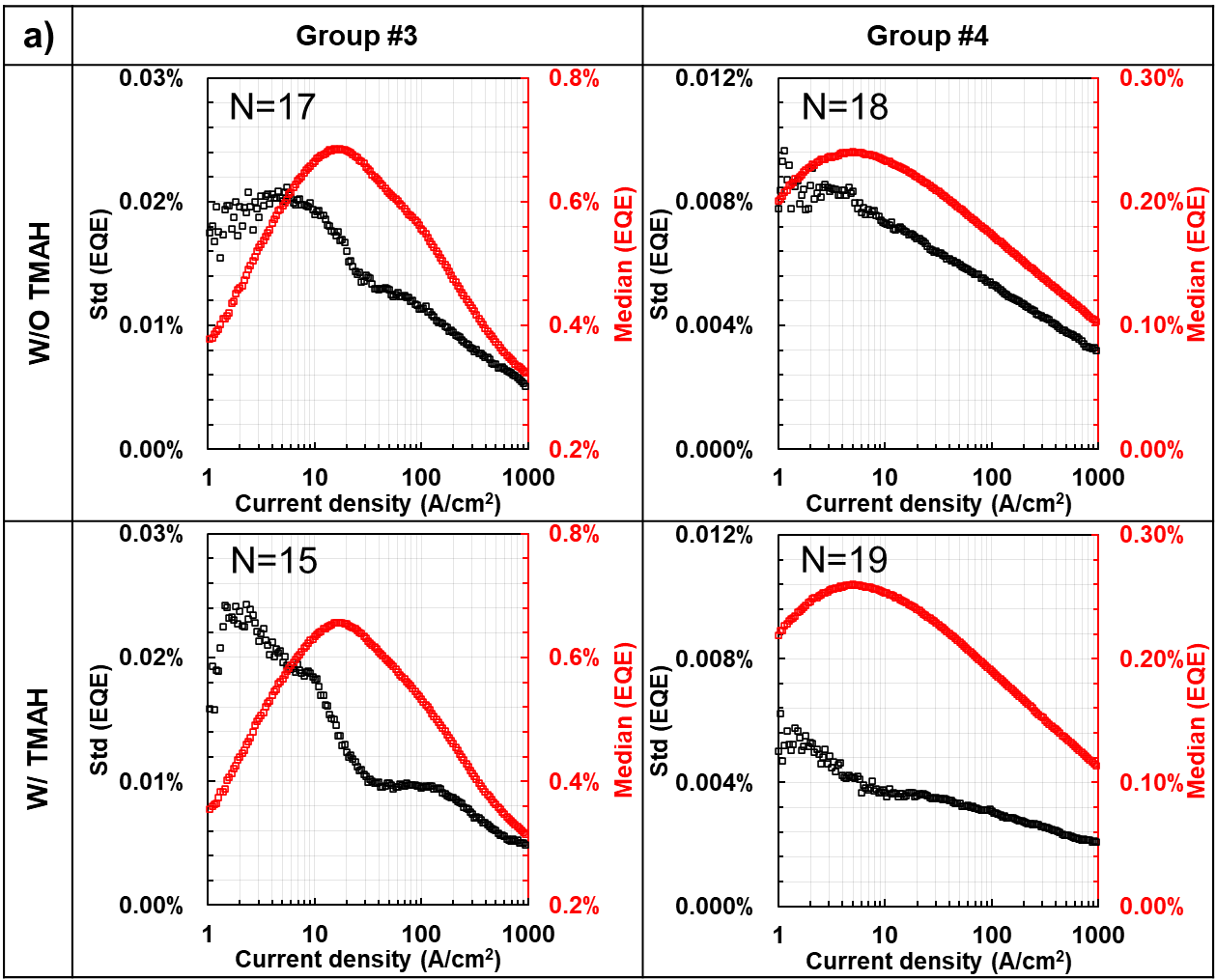


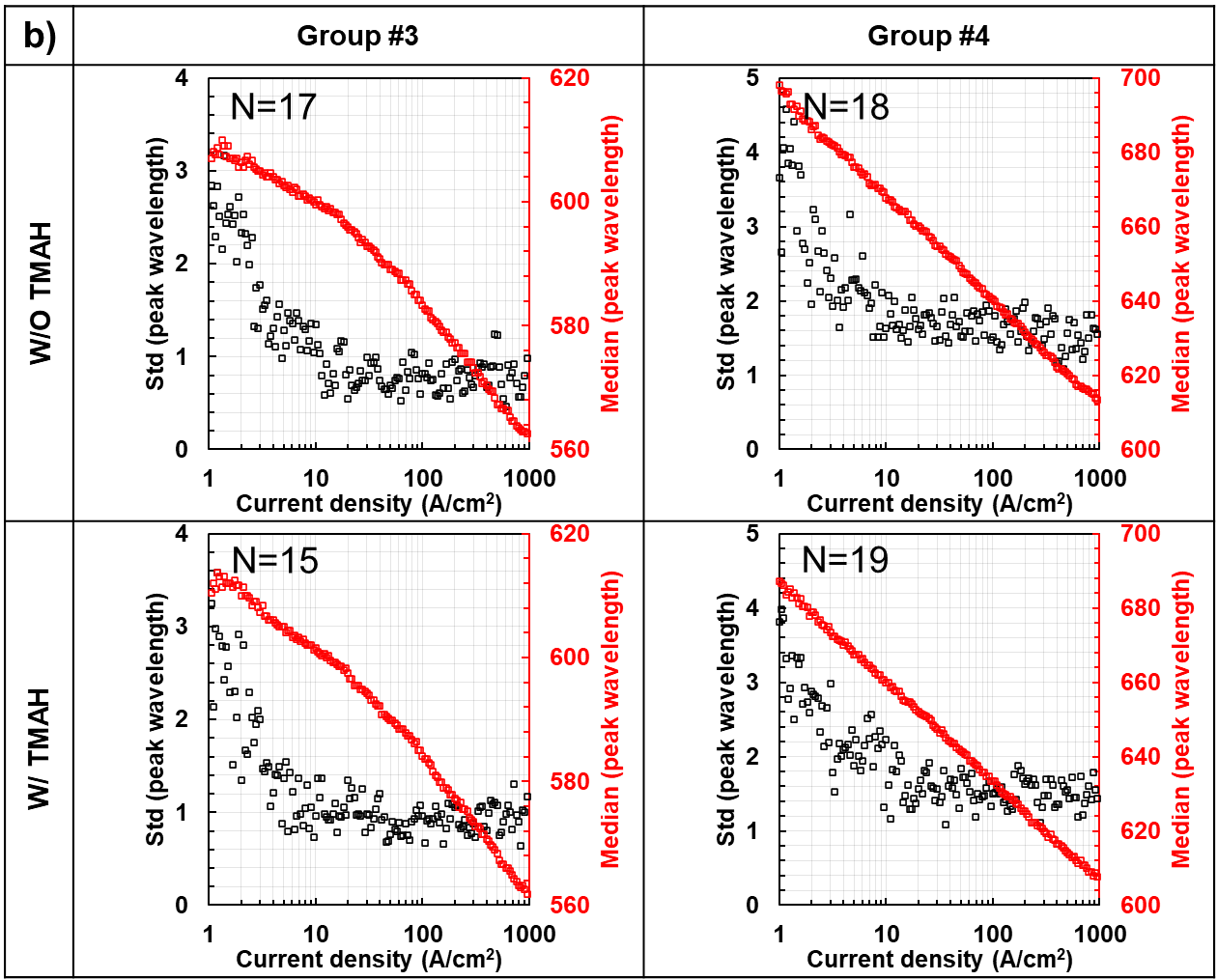


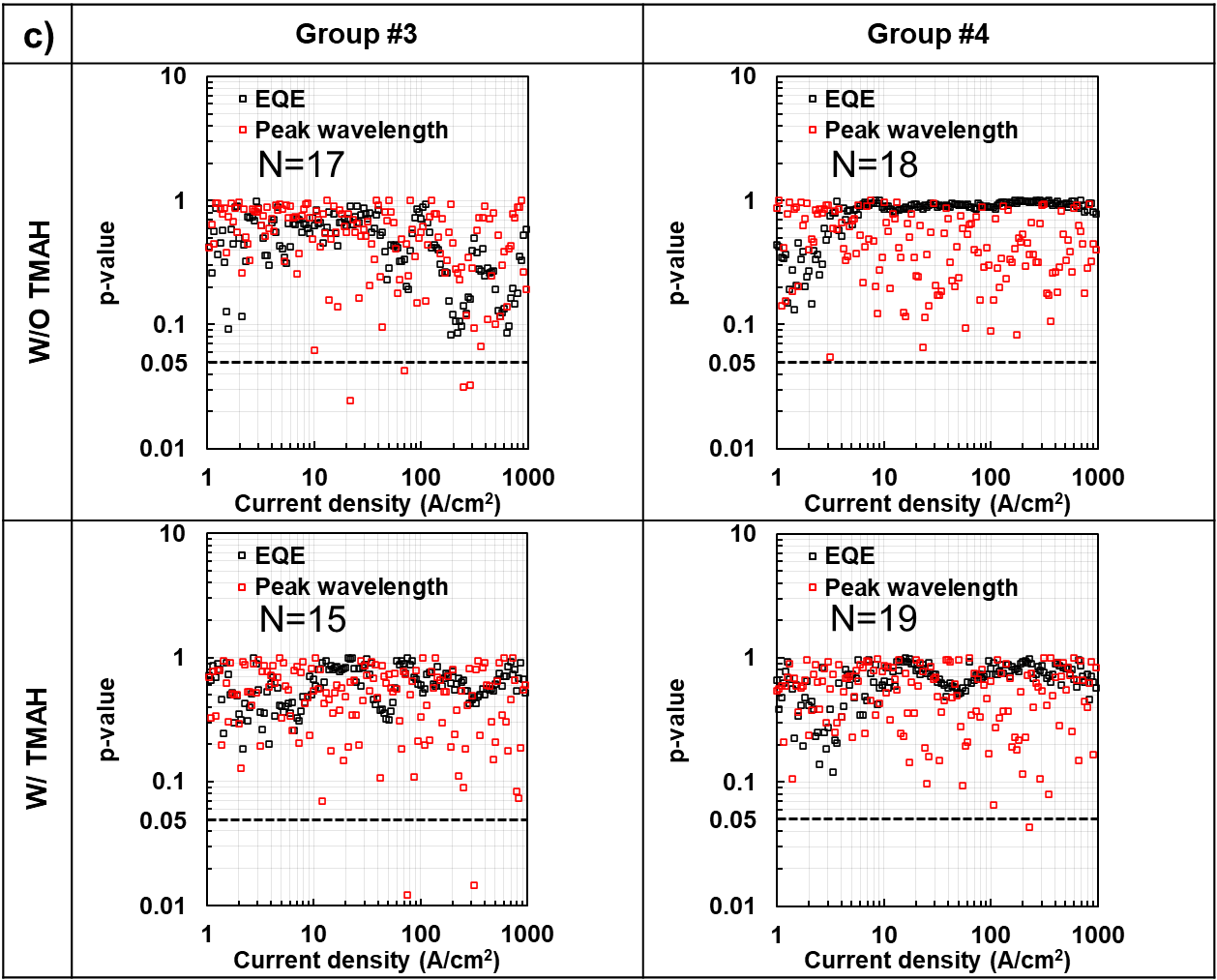


Figure S2. Standard deviation and median of EQE (a) and peak wavelength (b) for each group. c) p-value for each group. To calculate the p-value, the null hypothesis was that the average value is equal to the median value. Since most of the p-values at each current density are over 0.05, the null hypothesis cannot be denied, confirming the average value is not statistically different compared to the median value.
